# Propofol suppresses adipose-derived stem cell progression via PI3K/AKT-Wnt signaling pathway

**DOI:** 10.1186/s12871-022-01603-x

**Published:** 2022-03-09

**Authors:** Guoping Yin, Jia Wang, Yanling Zhong, Weidong Wu

**Affiliations:** 1 Department of Anesthesiology, The Second Hospital of Nanjing, Nanjing University of Chinese Medicine, Nanjing, 210003 China; 2 Department of Anesthesiology, Danyang People’s Hospital of Jiangsu Province & Danyang Hospital affiliated to Nantong University, Danyang, 212300 Jiangsu China

**Keywords:** Adipose-derived stem cells, Propofol, Proliferation, PI3K/AKT-Wnt

## Abstract

Adipose-derived stem cell (ADSC) transplantation has become a prospective way to treat cardiovascular diseases and skin traumas. Propofol, a short-acting intravenous anesthetic agent, plays an important role in the induction and maintenance of general anesthesia. In this study, we investigated the effects of propofol on ADSCs. The flow cytometry results showed that ADSCs were positive for CD29, CD44, and CD90 and negative for CD31, CD34, and CD45. The results of MTT and BrdU assays demonstrated that propofol impeded the proliferation of ADSCs. The cell scratch test showed that propofol had an inhibitory effect on the migration of ADSCs. Transwell assay showed that invasive ASDC counts decreased significantly after propofol treatment. Propofol also promoted ADSC apoptosis and arrested ADSCs in the G0/G1 phase. All these effects showed in a dose-dependent manner that the higher the concentration, the stronger the effect. Western blot analysis revealed decreased levels of FAK, PI3K, AKT, and GSK3β phosphorylation, while the phosphorylation of β-catenin increased after 48 h of treatment with propofol. The findings above indicated that the PI3K/AKT-Wnt pathways mediated propofol-inhibited ADSC proliferation, providing new insights into the propofol application in ADSCs.

**Supplementary Information:**

The online version contains supplementary material available at 10.1186/s12871-022-01603-x.

## Introduction

Adipose-derived stem cells generally originate from adipose tissues. Compared with bone marrow and umbilical cord blood, the adipose tissue is convenient to collect and has a higher cell yield/volume, making it a perfect source for adult stem cells [1]. ADSCs have self-renewal ability and multidirectional differentiation potential and can differentiate into skin, bone, nerve, myocardial, and other tissues [2, 3]. They also secrete many growth factors to promote skin repair and anti-aging processes and have important clinical applications in disease treatment and cosmetic medicine [4, 5]. Several studies have shown that certain adjuvant drugs can improve ADSCs when transplanted into target regions or organs. However, the therapeutic effect is not very clear due to low activity, low adhesion, low mobility, and low differentiation of transplanted cells [2, 6]. Propofol is a commonly used anesthetic agent in clinical practice. It has rapid effects, rapid reactions, and few adverse effects [7]. Recent studies have found that propofol can inhibit the proliferation of tumor cells and promote the apoptosis of tumor cells [8]. However, its possible role in the proliferation of ADSCs and the underlying mechanisms involved remain unclear.

Previous studies have shown that the phosphatidylinositol 3-kinase (PI3K)/−protein kinase B (AKT) pathway plays an important role in cell proliferation [9]. Moreover, Wnt signaling mainly affects the paracrine and autocrine functions of the cell membrane and integrates multiple signaling pathways, such as PI3K/AKT and glycogen synthase kinase (GSK3β) to mediate cell proliferation and other activities [10]. GSK3β is an essential regulator of various biological processes and signaling pathways, such as Wnt and PI3K/AKT [2, 10]. Furthermore, GSK3β is a key component of the typical Wnt signaling pathway, which degrades β-catenin through the phosphorylation and recruitment of ubiquitin proteasomes [11, 12]. After dephosphorization, β-catenin is transferred into the nucleus to interact with the T cytokine/lymphocyte enhancer factor-1 transcription factor family, resulting in the aberrant levels of genes associated with proliferation and differentiation [13]. Thus, this research aimed to explore the effect of propofol on ADSCs proliferation in vitro and determine whether the effect was associated with PI3K/AKT-Wnt pathways.

## Materials and methods

### Isolation and culture of ADSCs

ADSCs were isolated and cultured from adipose tissues from male mice (C57BL/6) at 3 weeks of age, that were purchased from Cavens (Changzhou, China). First, these mice were euthanized by intraperitoneal injection of sodium pentobarbital at dose of 200 mg/kg, then adipose tissues were separated from the inguinal area. Next, visible blood vessels, lymph nodes, and fascia were removed. The cut tissues were added to 1.25%W/V papain and shaken gently at 37 °C for 30 min. The floating adipocytes were separated by papain neutralization and centrifuged at 1000 rpm for 5 min at 4 °C. The resuspended cells were cultured in low glucose (DMEM; Gibco, USA), 10% fetal bovine serum (FBS; Gibco, USA), 100 U/mL penicillin, 0.1 mg/mL streptomycin (Thermo Fisher Scientific, USA), 10 ng/mL vascular endothelial growth factor (VEGF), 10 ng/mL basic fibroblast growth factor (FGF), and alpha FGF (Sigma Aldrich, St. Louis, Missouri, USA) at 37 °C and 5% CO_2_. All the animal experiments were approved by the Animal Ethics Committee of the Nanjing University of Chinese Medicine, performed under the Guide for the Care and Use of Laboratory Animals of the National Institutes of Health, and reported under ARRIVE guidelines.

### Flow cytometry

The immunophenotype of ADSCs was analyzed by flow cytometry, including the expression of CD29, CD31, CD34, CD44, CD45, and CD90. Surface antigens can be detected by collecting third-generation adipose stem cells. After washing twice in PBS at 4 °C, CD29 (10 μg/mL, EBioscience,11–0291-82), CD31 (1:50, Invitrogen, MA1–80069), CD34 (1:50, Invitrogen, PA5–85917), CD44 (1:50, Invitrogen MA5–16908), CD45 (5 μg/mL EBioscience, 17–0461-82), and CD90 (0.6 μg/mL, EBioscience, 17–0900-82) were labeled with anti-mouse FITC-polyclonal antibodies. The cells were collected in cold PBS and analyzed by flow cytometry (FACSA ria TM, BD, USA).

### Cell viability measurement

The 3-(4,5-dimethylthiazol-2-yl)-2,5-diphenyltetrazolium bromide (MTT) assay was used to detect cell proliferation [14]. Each group was set up with three parallels. Propofol was purchased from MedChemExpress (No. HY-B0649). The cells were digested and pipetted into a single cell suspension, counted, and inoculated in a 96-well plate at 4000 cells/well. After culturing with different propofol concentrations (0, 2.5, 5, 10, 15, 20, 30, 40, 60, and 80 μM) on the seed plate for 12, 24, 48, and 72 h, the culture solution was discarded. After washing twice with PBS, 10-μL of MTT solution (5 g/L) was added to the wells and incubated again for 4 h. After discarding the MTT solution, 150 μL of DMSO was added to each well, followed by incubation at a constant temperature for 10 min. The absorbance value of each well in each group was measured at a wavelength of 490 nm in the immune detector.

### BrdU assay

ADSCs were set up in the control group (0.1% DMSO) and the propofol groups (low, medium, and high) and co-incubated with 10-μM bromodeoxyuridine (BrdU, Abcam, ab8152) for 40 min at 37 °C. The cells were immobilized, acidified, and then incubated with an anti-BrdU antibody (1:250, Abcam, ab6326) to detect the content of BrdU in DNA. Then the cells were followed by DAPI staining for 10 min. Quantitative studies were based on five random fields by confocal microscopy.

### Cell scratch experiment

After 24 h of culture, the cells in each group were inoculated in a 12-well culture plate at 2 × 10^8^/mL. When the fusion degree reached 90%, 10 μL of sterile spearhead was used to scratch the straight line along the bottom of the culture plate to form a gap. After washing with PBS three times and adding the serum-free medium, the culture continued in an incubator. After 48 h, the cells’ healing degree was observed under a microscope and photographed, and the area between cells was measured. The wound healing percentage was calculated using the following formula: wound healing (%) = [1- the scratch area (48 h) / the scratch area (0 h)] × 100%.

### Transwell assay

The top chamber of the Transwell plate was pretreated with 300 μL of a serum-free medium and incubated for 2 h at room temperature. Each group of cells was suspended in the serum-free medium and diluted to 5 × 10^5^ cells/mL; 300 μL of the cell suspension was added to the top chamber of the Transwell plate with or without propofol, and the lower chamber was filled with 500 μL of the medium containing FBS. After 24 h of incubation at 37 °C, the inserts were stained with 0.1% crystal violet for another 20 min. After washing, five fields (× 200) of the upper chamber’s lower layer were selected randomly to observe and count the invading cells under a microscope.

### Cell apoptosis and cell cycle assay

Flow cytometry was conducted to study the effect of propofol on the apoptosis and cell cycle. The Annexin V-FITC Apoptosis Detection Kit and Cell Cycle Detection Kit were provided by Key GEN Bio TECH (Nanjing, China). The assay was carried out based on the manufacturer’s protocol.

### Western blot analysis

Western blotting was applied to investigate the expression of ADSC protein related to PI3K-AKT/Wnt-β-catenin signaling pathways. The total proteins of the cells in each group were extracted by Ripa lysate and quantified by the BCA method. The samples were denatured and loaded. Electrophoresis was carried out according to the experimental steps, one-turn film sealing (5% skimmed milk powder), incubation with primary antibody (4 °C, overnight), incubation with secondary antibody (room temperature, 1 h), and development exposure. The gray value of protein bands was analyzed by the ProPlus-image analysis system. The primary antibodies included FAK (5 μg/mL, Invitrogen, PA5–16676), P-FAK (1:2000, Invitrogen, OPA1–03887), PI3K (1:500, Invitrogen, MA1–74183), P-PI3K (1:500, Invitrogen, MA1–104853), AKT (1:10000, Abcam, ab179463), p-AKT (1:500, Abcam, ab38449), Wnt3a, GSK3β (1:1000, Abcam, ab93926), p-GSK3β (1:500, Abcam, ab75745), β-catenin (1:400, Abcam, ab224803, p-β-catenin (1:500, Abcam, ab757777), cyclinD1 (1:10000, Abcam, ab134175), and GAPDH (1:10000, Abcam, ab181602). The secondary antibody was goat anti-rabbit IgG antibody (1:4000, Abcam, ab6721)). GAPDH was considered a control, and the gray value of the protein band was analyzed by the Proplus image analysis system. Because some blots were cut prior to hybridization with antibodies, the image results showed the absence of adequate length. All replicates of blot results by western blot have been provided in the Supplementary Information.

### Statistical analysis

The measurement data were expressed by mean ± standard deviation. Graphpad Prism software (version 6.0) was used for statistical analyses. The statistical significance between the groups was calculated using s Student’s t-test, ANOVA, or [the Student-Newman-Keuls (SNK) test for the post hoc tests] if the data were normally distributed. Otherwise, they were analyzed using the Kruskal-Wallis test (K-W test). *P* < 0.05 indicated a significant difference.

## Results

### Detection of surface markers of ADSCs

**Fig. 1:**
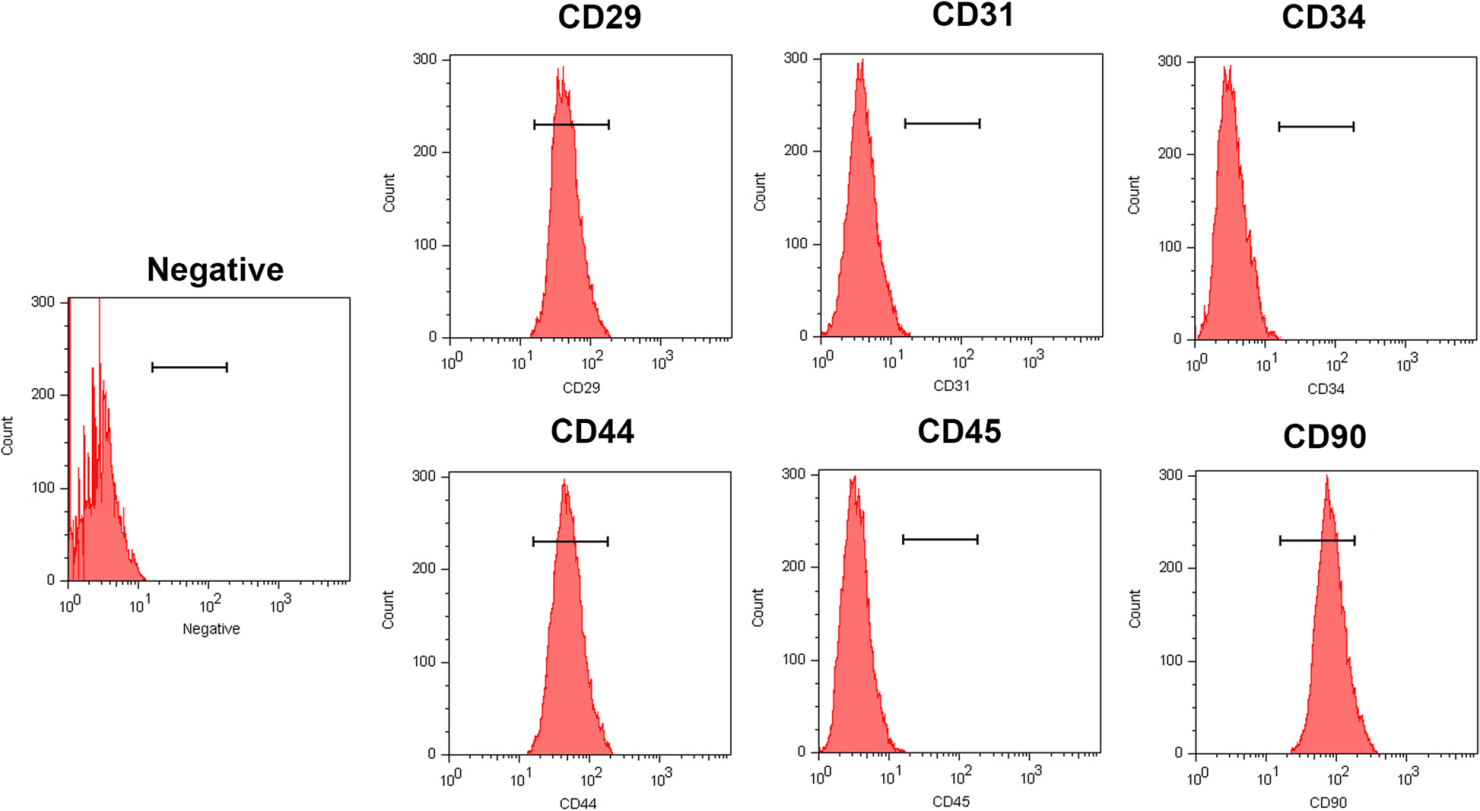
Characterization analysis of ADSCs by flow cytometry. ADSCs were positive for mesenchymal stem cell surface antigen CD29, CD44 and CD90, but negative for hematopoietic surface antigen CD34 and CD45, and also negative for vascular endothelial cell antigen CD31

According to the phenotype analysis by flow cytometry (Fig. 1), after three rounds of culture, ADSCs were positive for and highly expressed stem cell surface antigens CD29, CD44, and CD90, but negative for CD31, CD34, and CD45. Therefore, we demonstrated that the cells originated from adipose tissue and had representative ADSC features.

### Effect of propofol on ADSC proliferation

**Fig. 2:**
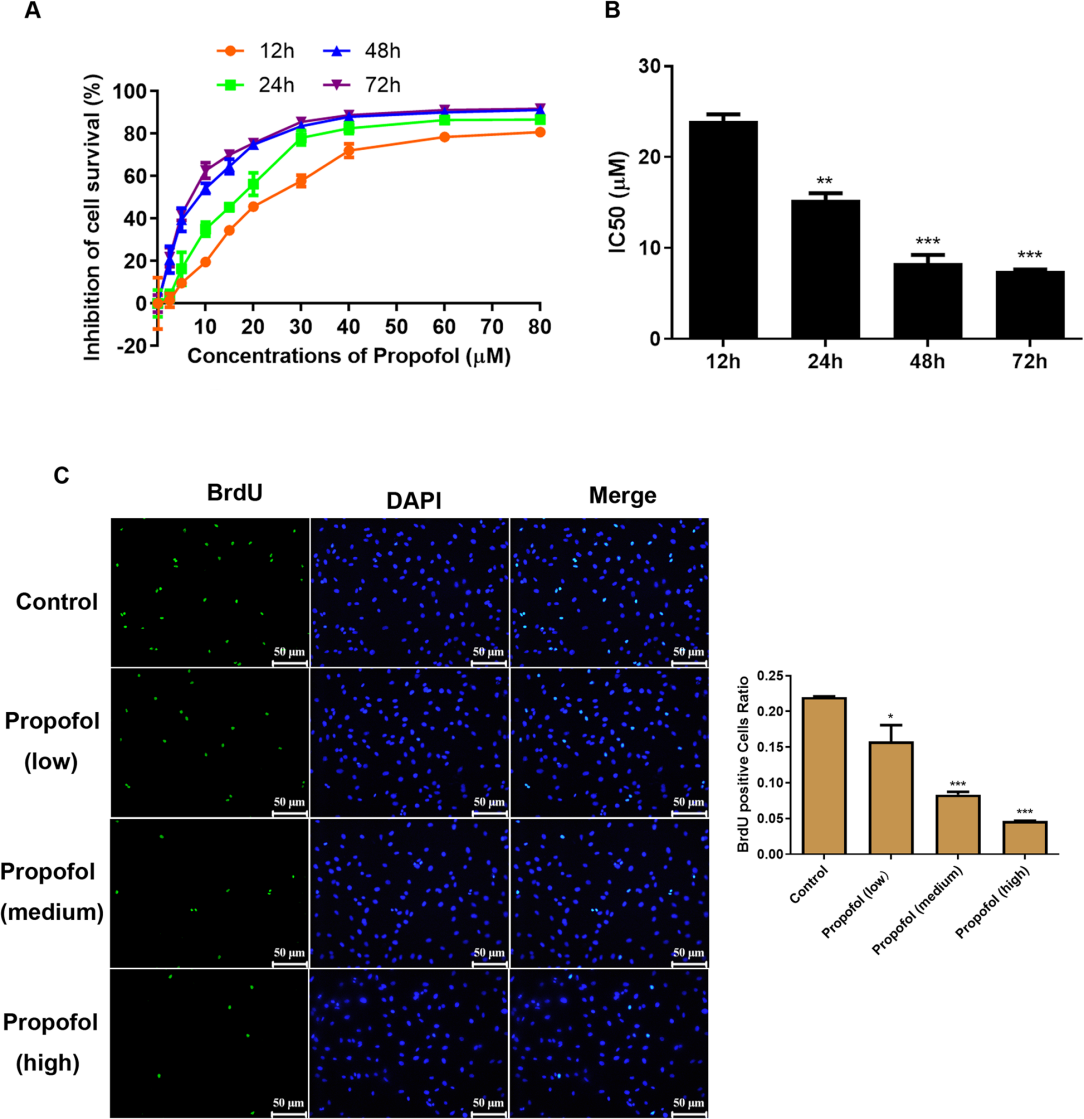
The effect of propofol on the proliferation of ADSCs. **A** The MTT result of propofol at different time and concentrations in ADSCs; **B** The IC50 value calculated from (A), the final selection time is 48 h, and the IC50 corresponding to 48 h is 8.3 μM (1/5 IC50 as low concentration, 1/3 as medium concentration and 1/2 as high concentration, that is 1.7 μM, 2.8 μM and 4.2 μM respectively). **C** The results of BrdU assay. Values are expressed as mean ± SD (*n* = 5). *p < 0.05, **P < 0.01 vs. control

To investigate the effect of propofol on the biological function of adipocytes, an MTT assay was applied to detect the cell viability in the 2.5–80 μM range. As shown in Fig. 2A, the use of different propofol concentrations would significantly inhibit the growth of ADSCs after different incubation times: the higher the concentration, the longer the incubation time, the lower the viability.

According to the above results, IC50 values were calculated as shown in Fig. 2B. The final incubation time was 48 h, and the corresponding IC50 was 8.3 μM. Based on this IC50 concentration, 1/5 IC50 was a low concentration, 1/3 was a medium concentration, and 1/2 was a high concentration, respectively: 1.7, 2.8, and 4.2 μM.

BrdU was also applied to test the proliferation ability of the cells. As shown in Fig. 2C, propofol significantly inhibited the proliferation of ADSCs: the higher the concentration, the lower the positive ratio. The above results confirmed that propofol had a significant inhibitory effect on ADSCs.

### Effect of propofol on the migration of ADSCs

**Fig. 3:**
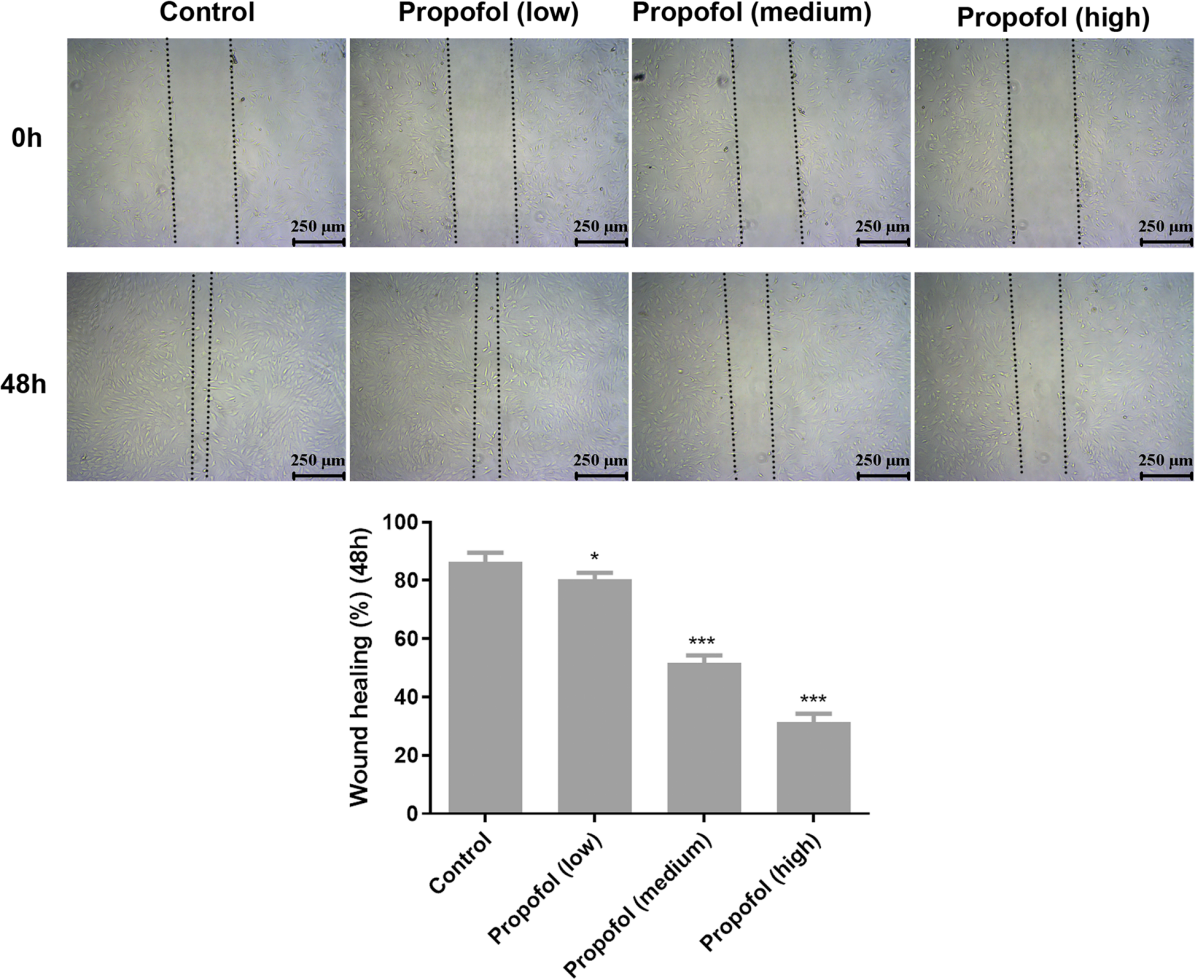
The effect of propofol on migration ability of ADSCs by scratch test. The low, medium and high concentrations were 1.7 μM, 2.8 μM and 4.2 μM respectively. Values are expressed as mean ± SD (*n* = 3). *p < 0.05, **P < 0.01 vs. control

The results of the cell scratch experiment (Fig. 3) showed that, compared with the control group, propofol affected ADSC migration, and the effect of high concentration was more evident than that of low and medium concentrations, indicating a concentration-dependent pattern.

### Effect of propofol on the invasion of ADSCs

**Fig. 4:**
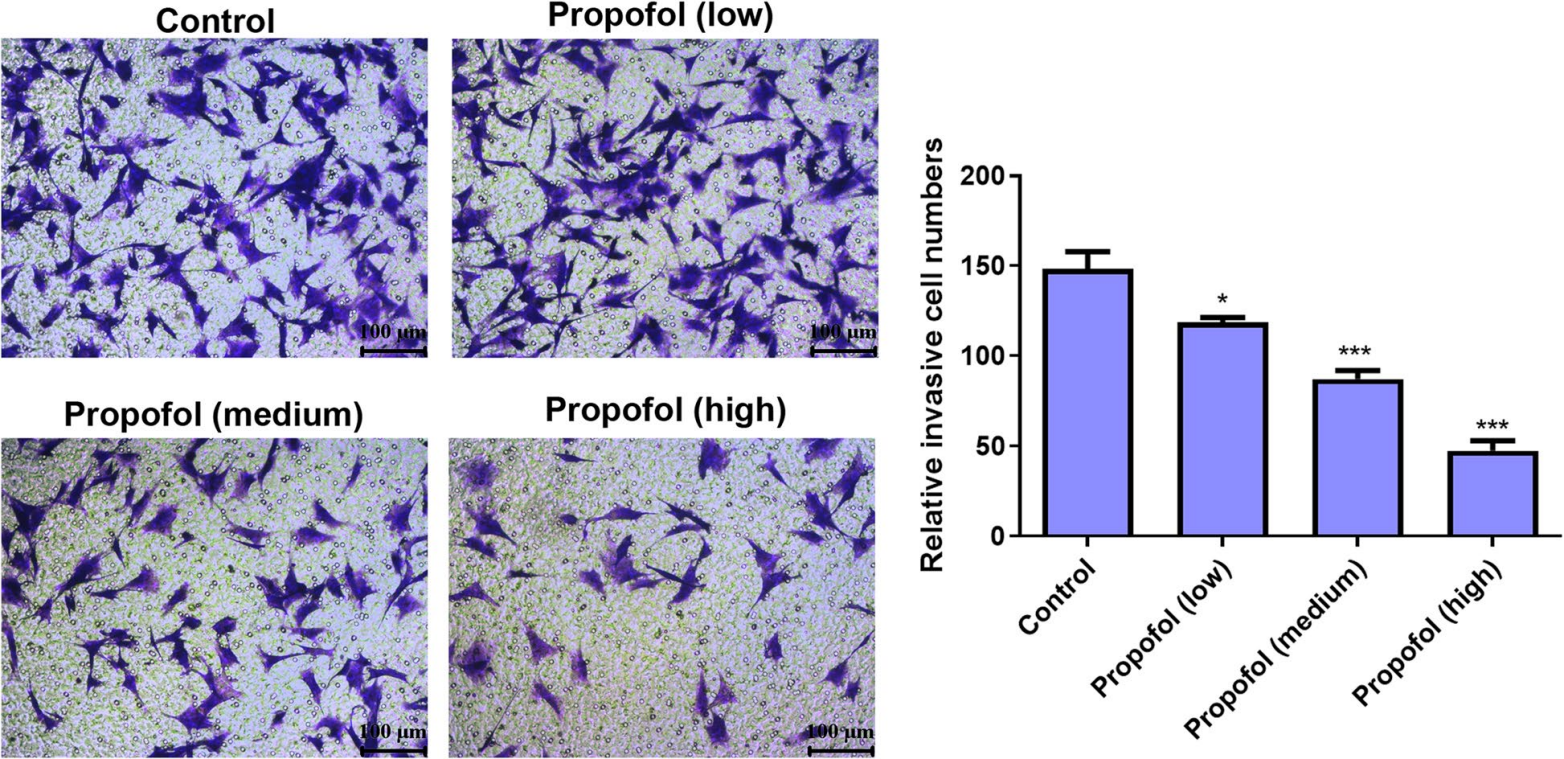
The effect of propofol on invasion ability of ADSCs by Transwell assay. The low, medium and high concentrations were 1.7 μM, 2.8 μM and 4.2 μM respectively. Values are expressed as mean ± SD (*n* = 5). *p < 0.05, **P < 0.01 vs. control

Figure 4 presents the Transwell test results; compared with the control group, the number of cells per unit area decreased after propofol treatment. In addition, the number of cells in high concentrations was significantly lower than those in medium and low concentrations, indicating a concentration-dependent pattern.

### Effect of propofol on the apoptosis of ADSCs

**Fig. 5:**
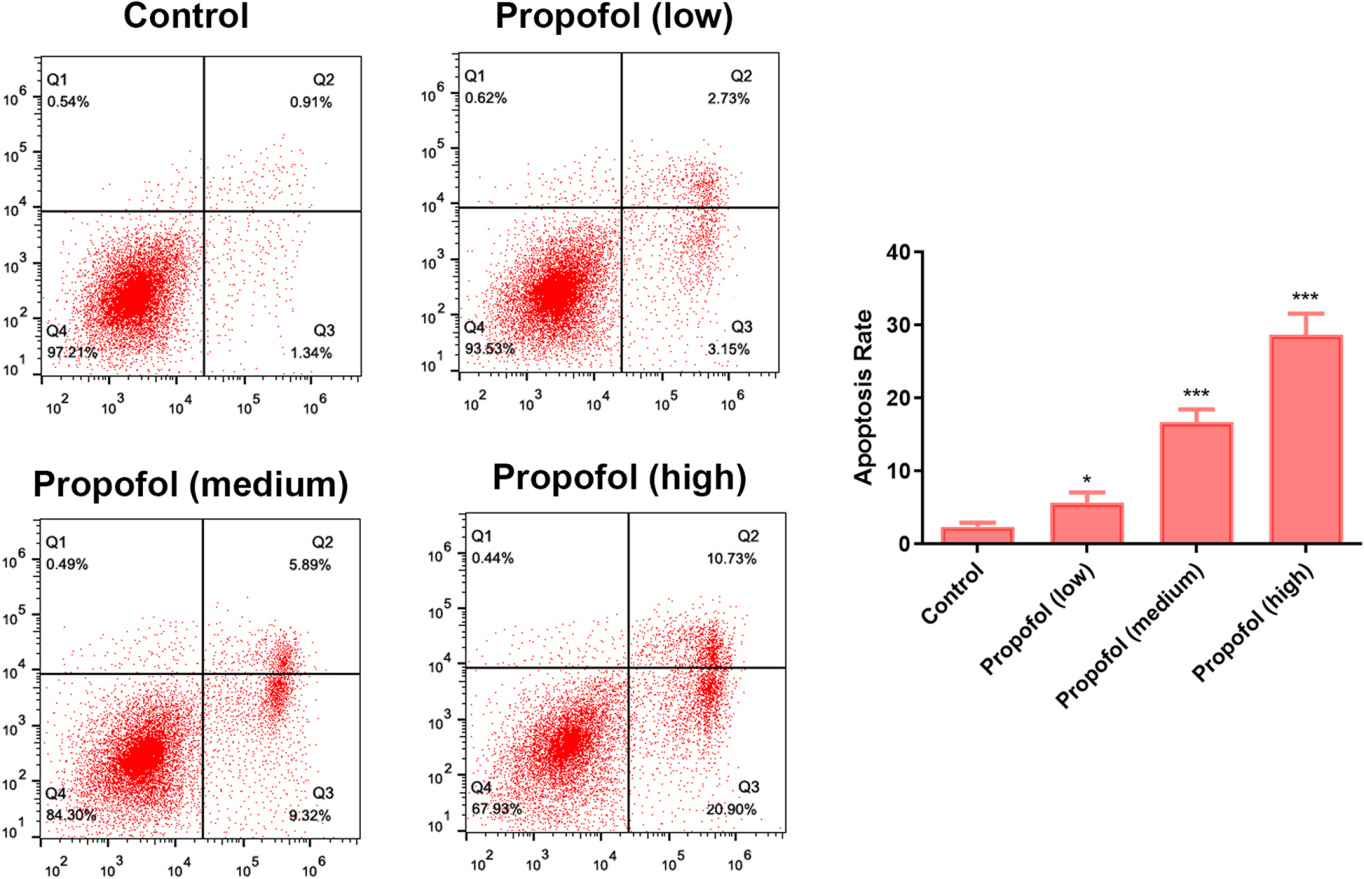
The effect of propofol on apoptosis of ADSCs by flow cytometry. The low, medium and high concentrations were 1.7 μM, 2.8 μM and 4.2 μM respectively. Values are expressed as mean ± SD (*n* = 3). *p < 0.05, **P < 0.01 vs. control

Flow cytometry results showed that the apoptosis rate (the upper right quadrant plus the low right quadrant) of cells treated with propofol was significantly higher than that in the control group (Fig. 5). In addition, the number of apoptotic cells at high concentrations was significantly higher than that at medium and low concentrations.

### Effect of propofol on the cell cycle of ADSCs

**Fig. 6:**
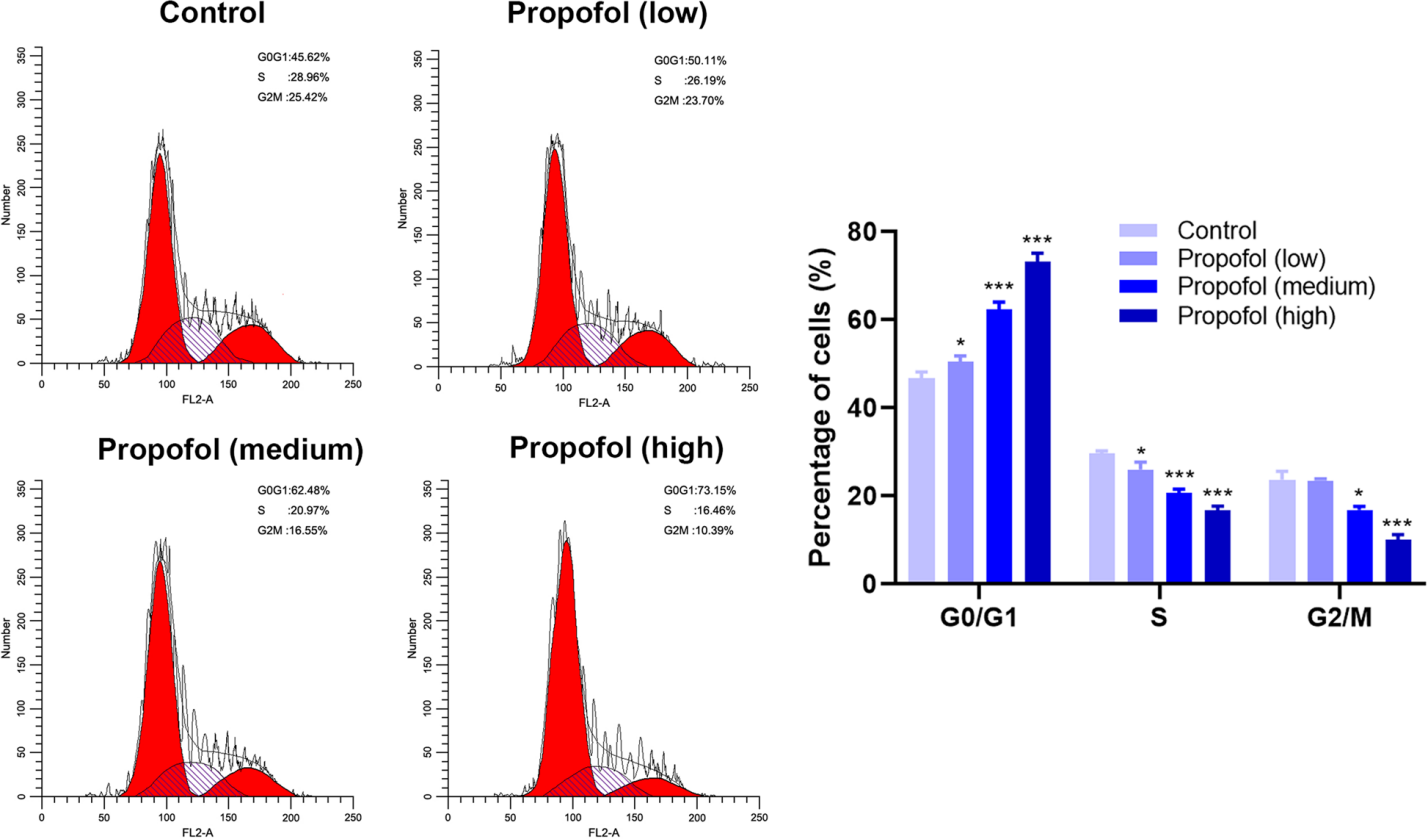
The effect of propofol on cell cycle of ADSCs by flow cytometry. The low, medium and high concentrations were 1.7 μM, 2.8 μM and 4.2 μM respectively. Values are expressed as mean ± SD (*n* = 3). *p < 0.05, **P < 0.01 vs. control

According to the results of the flow cycle experiment (Fig. 6), compared with the control group, the number of cells in the G0/G1 phase after propofol treatment was higher, and the number of G0/G1 cells in high concentration was higher than that in medium and low concentrations. Propofol treatment arrested ASSCs in the G0/G1 phase, blocking the transition to the S phase.

### Effect of propofol on the PI3K/AKT and Wnt signaling pathways

**Fig. 7:**
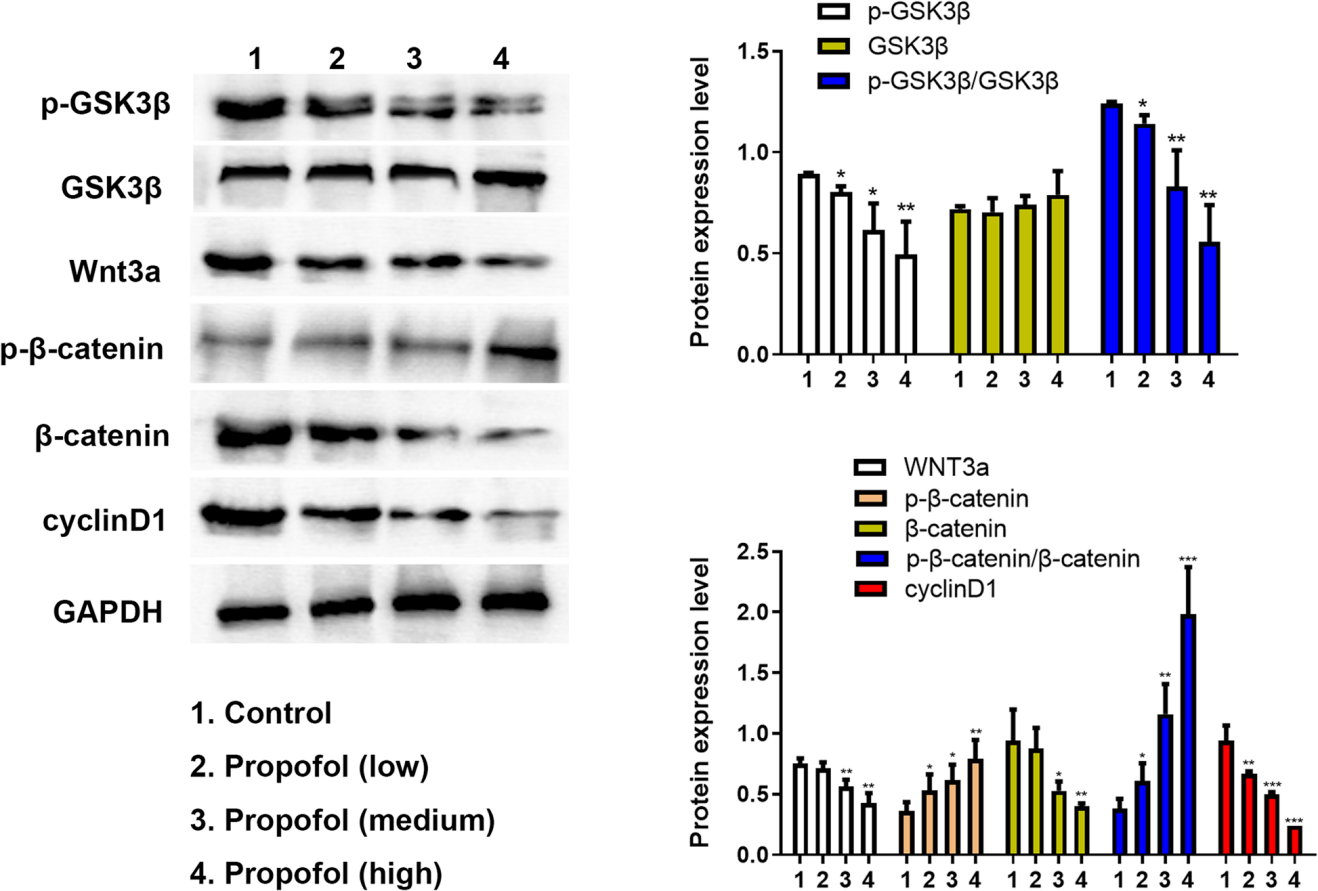
The effect of propofol on p-GSK3β, GSK3β, Wnt3a, p-β-catenin, β-catenin and cyclinD1 in ADSCs by western blot and the quantitative analysis. The quantitative results were standardized according to GAPDH level. The low, medium and high concentrations were 1.7 μM, 2.8 μM and 4.2 μM respectively. Values are expressed as mean ± SD (*n* = 5). *p < 0.05, **P < 0.01 vs. control

**Fig. 8:**
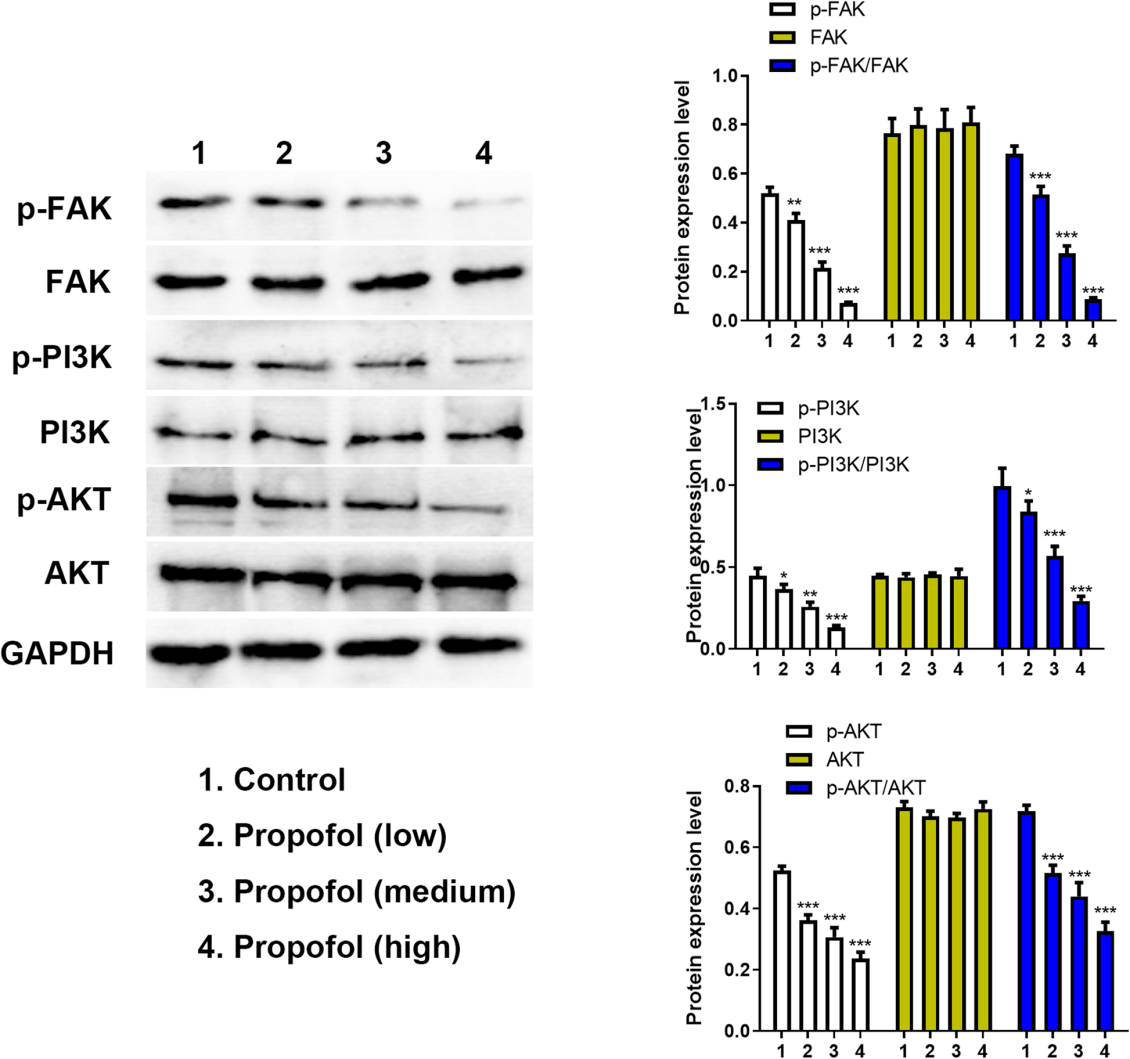
The effect of propofol on PI3K/AKT signaling in ADSCs by western blot and the quantitative analysis, including p-FAK, FAK, p-PI3K, PI3K, p-AKT and AKT. The quantitative results were standardized according to GAPDH level. The low, medium and high concentrations were 1.7 μM, 2.8 μM and 4.2 μM respectively. Values are expressed as mean ± SD (*n* = 3). *p < 0.05, **P < 0.01 vs. control

It has been reported that propofol inactivates the p38 MAPK signaling pathway [15]. In addition, ADSC proliferation has been demonstrated to be closely associated with PI3K and Wnt pathways [16, 17]. GSK3β influences various pathways, including Wnt and PI3K/AKT, which is considered a vital regulator of diverse biological processes. As a result, the levels of phosphorylated GSK3β, Wnt signaling pathway, GSK3β, Wnt’s downstream target proteins, and also PI3K/AKT pathway were determined by western blotting. As shown in Fig. 7, compared with the control group, propofol could significantly control the phosphorylation of GSK3β, reduce the expression of WNT3a and cyclin D1, and contribute to the remarkable increase in p-β-catenin and a decrease in β-catenin in a dose-dependent manner. These findings suggest that propofol inactivated β-catenin and reduced Wnt3a and cyclinD1 of the Wnt signaling pathway in ADSCs. Concerning the PI3K/AKT signaling pathway in Fig. 8, the levels of p-PI3K, p-FAK and, and p-AKT decreased steadily in ADSCs compared to the control group. However, the primary levels of FAK, PI3K, and AKT did not change. These findings indicated that the PI3K/AKT pathway was inactivated by propofol treatment in ADSCs.

## Discussion

It has been confirmed by previous research that ADSCs are adult stem cells with pluripotent differentiation potential extracted from the adipose tissue [18]. ADSCs have great application potential in treatment, rehabilitation, and cosmetology, as the largest adult stem cell bank in human tissue engineering, and will become an ideal cell source in the field of stem cell applications [19]. Propofol, the most widely used intravenous anesthetic agent, is characterized by a fast onset, strong action, and quick recovery. However, it has many complications, such as apnea and blood pressure. Propofol’s mechanism of action is mainly by activating the Y-aminobutyric acid receptor, opening chloride channels, resulting in the influx of chloride ions, intracellular hyperpolarization, and post-synaptic inhibition, thereby reducing central excitability [20, 21]. Propofol, used to induce general anesthesia, can decrease the patient’s blood pressure and even decrease oxygen consumption and peripheral cardiovascular resistance, inducing the inhibitory effect on the circulatory system [22, 23]. This study revealed the effect of propofol on ADSC proliferation, migration, and apoptosis.

Based on the results of MTT experiments in the present study, propofol can inhibit the proliferation of ADSCs when the concentration of propofol is > 5 μM. The IC50 concentration of propofol at 48 h was selected as the benchmark: 1/5 of IC50 was considered low concentration, 1/3 was a medium concentration, and 1/2 was a high concentration in the 48-h culture, indicating that the proliferative ability of ADSCs was significantly reduced after propofol treatment in a concentration-dependent pattern. The BrdU experiment was applied to further explore cell proliferation at different concentrations. It also indicated that propofol suppressed ADSC proliferation. The scratch assay, Transwell assay, and flow cytometry assay demonstrated that propofol inhibited migration and invasion and promoted apoptosis of ADSCs.

Based on previous research, the Wnt signaling pathway is a key factor in promoting the survival and growth of various cells [24]. In the Wnt signaling pathway, by inhibiting GSK3β, the nucleus accumulates to form complexes with the TCF/LEF family to regulate the expression of specific downstream genes required for cell proliferation, including cyclinD1 [11, 25]. An important regulator of Wnt is a secretory protein that functions through autocrine or paracrine systems [26]. After secretion, Wnt can interact with specific receptors on the cell surface through a whole train of phosphorylation and depletion of downstream proteins [27, 28]. The phosphorylation process causes β-catenin to accumulate, which is a multi-purpose protein that interacts with E-cadherin at cell junctions and participates in the process of adhesion bands [29]. Free β-catenin can enter the nucleus and affect the expression of target genes [30]. GSK3β is a serine/threonine protein kinase. Studies have also shown that Wnt integrates with other signaling pathways, including the PI3K/Akt pathway. PI3K is a dimer composed of regulatory subunit p85 and catalytic subunit p110 [31], the main signaling pathway involved in cell growth, proliferation, and transformation [32]. AKT is a vital component of the PI3K/AKT signaling pathway [33]. When PI3K combines with growth factor receptors, it can affect Akt protein structure and activity, and through phosphorylation, it can activate or inhibit a series of downstream substrates, including apoptosis-related proteins Bad and Caspase9 [34]. Therefore, it can be concluded that by adjusting the PI3K/AKT and Wnt pathways, the biological characteristics of cells can be controlled [35, 36].

Therefore, we continue to study the cell signaling mechanism for the inhibitory effect of propofol on ADSCs to determine whether propofol inhibits the proliferation, migration, and movement of ADSCs and promotes cell apoptosis by affecting PI3K/Akt and Wnt/β-catenin pathways.

Western blotting showed that the phosphorylation of GSK3β, FAK, PI3K, and AKT decreased, while the phosphorylation of β-catenin was enhanced. In addition, the expression of Wnt3a, β-catenin, and cyclinD1 was significantly reduced. These findings suggest that PI3K/Akt and WNT/β-catenin pathways are inhibited. It was concluded from these findings that propofol suppressed the proliferation of ADSCs by inactivating PI3K/AKT and WNT/β-catenin signaling pathways. This study suggested that propofol should be used in the clinic judiciously due to its side effect on the normal cells, including ADSCs. Furthermore, when the patients are treated with ADSC transplantation, the dose of propofol should be more specific to avoid toxicity to ADSCs.

## Conclusion

In summary, this study revealed that propofol inhibited the proliferative ability of ADSCs, necessitating the careful clinical application of propofol in ADSC transplantation procedures.

## Supplementary Information

**Additional file 1.**

## Data Availability

The data of this study could be requested from the corresponding authors.
