# Supplementary material for: Propofol suppresses adipose-derived stem cell progression via PI3K/AKT-Wnt signaling pathway
Source: BMC Anesthesiol. 2022 Mar 9;22:65. doi: 10.1186/s12871-022-01603-x (PMC8905820; doi:10.1186/s12871-022-01603-x)
Supplement: Supplementary file 1 — Additional file 1. [file 12871_2022_1603_MOESM1_ESM.pdf]

The original gels of Fig.7 in the first experiment:

p-GSK3β

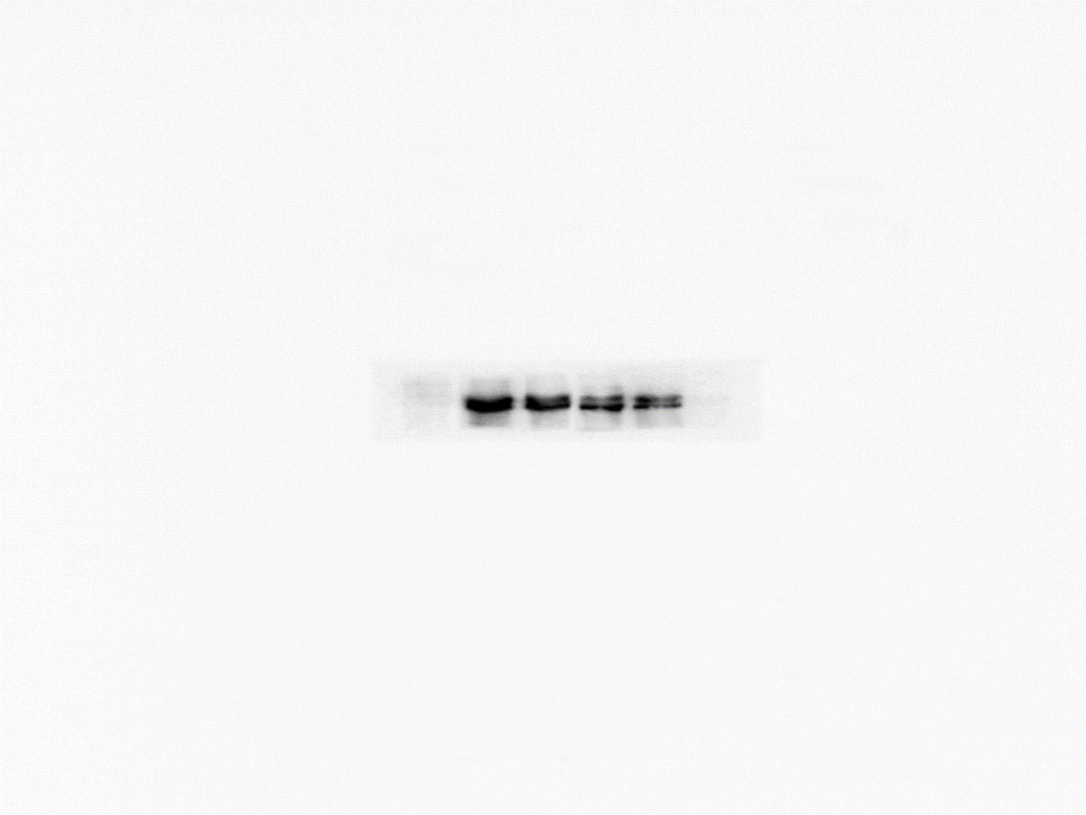

GSK3β

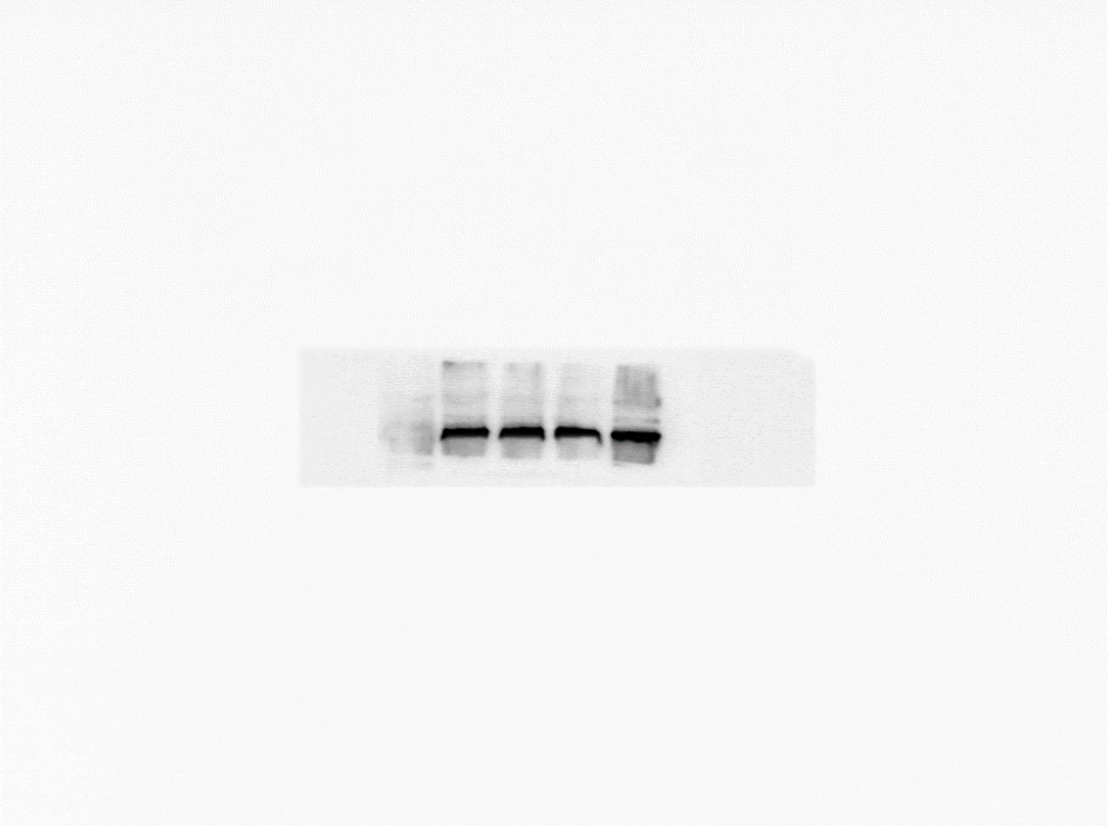

Wnt3a

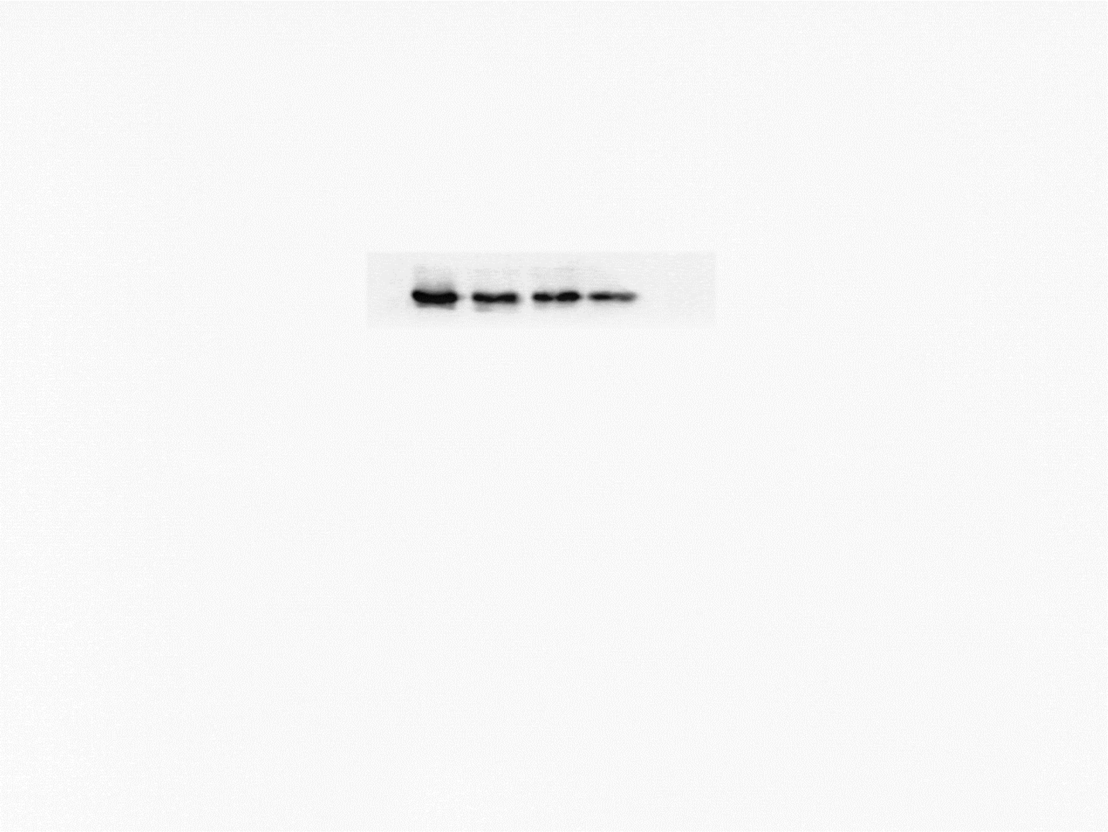

p-β-catenin

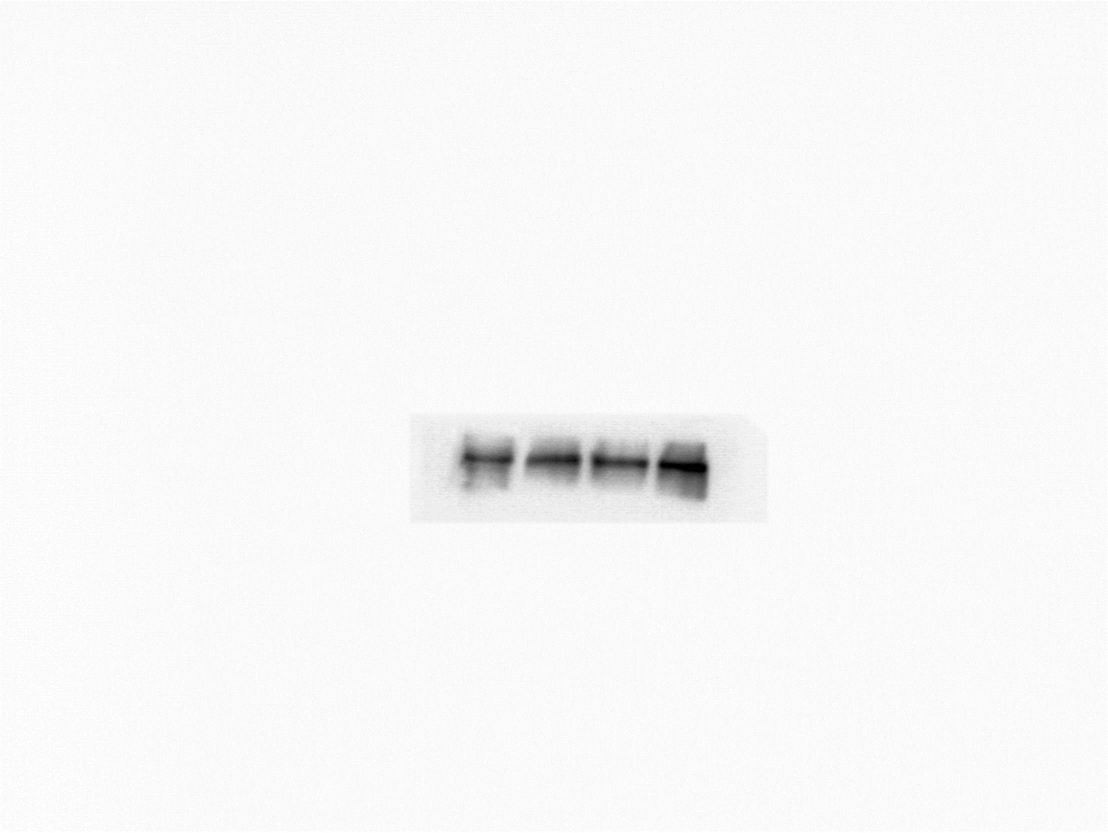

β-catenin

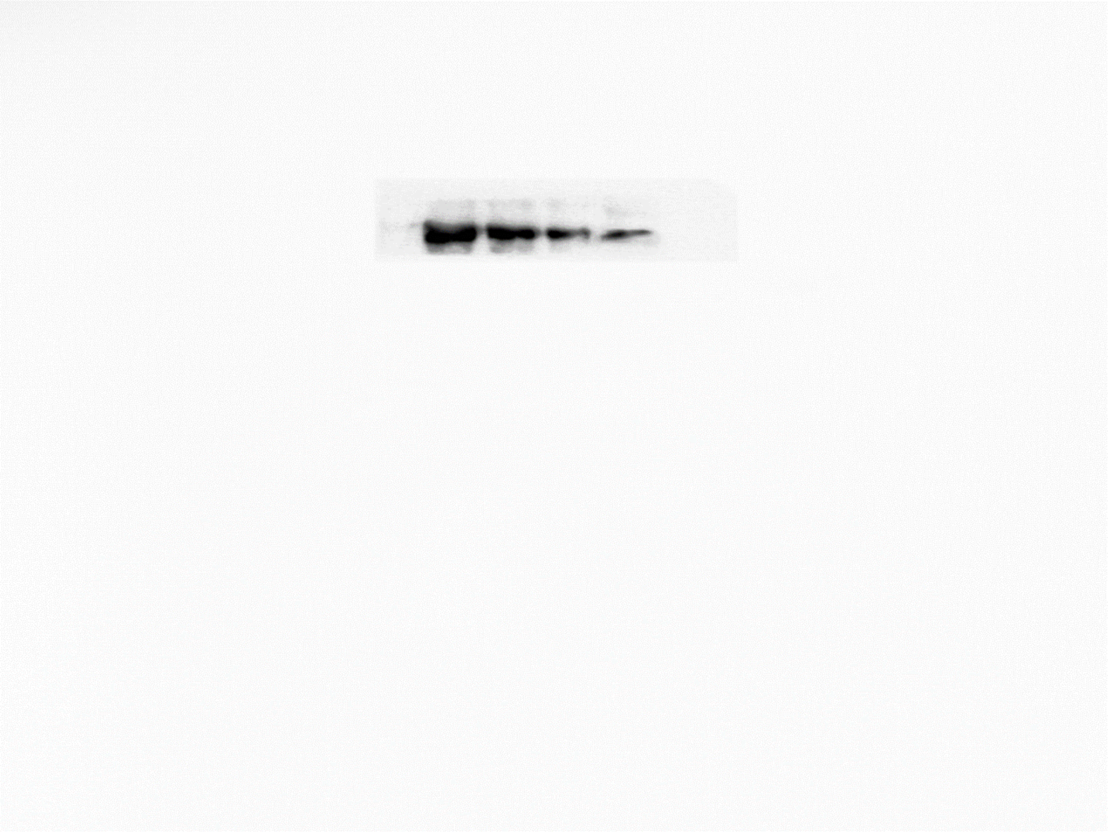

cyclinD1

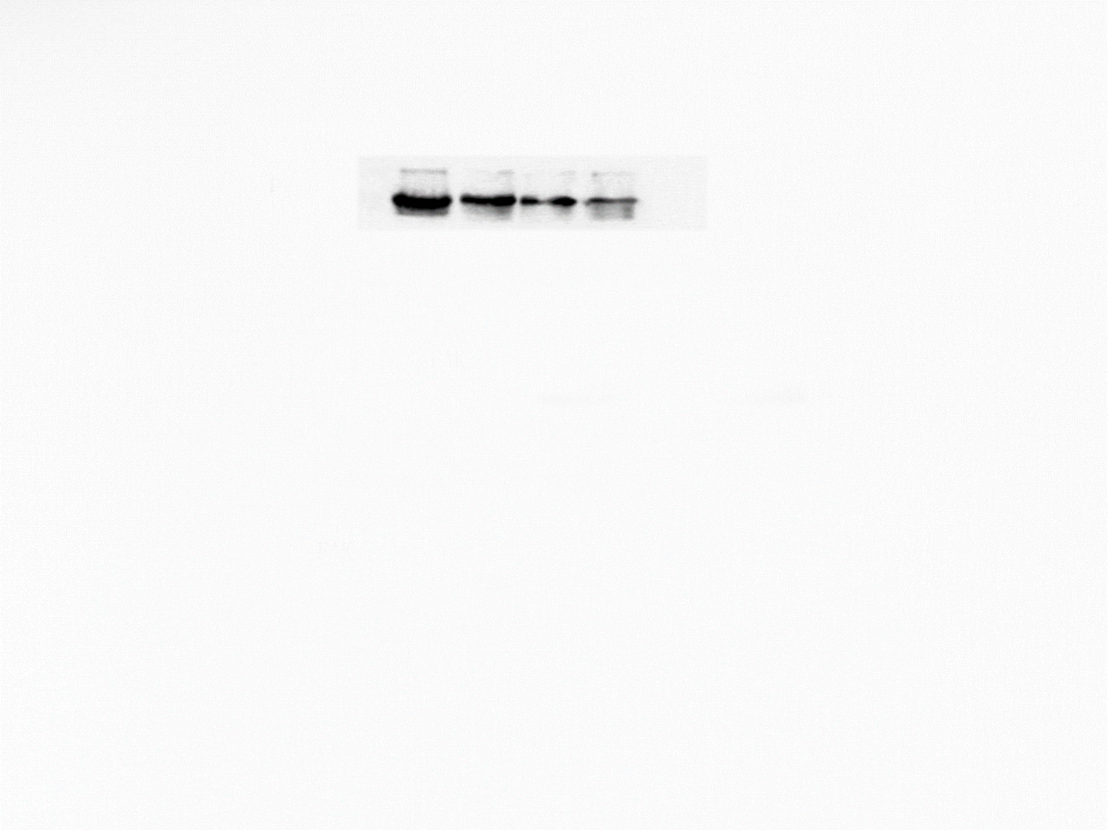

GAPDH

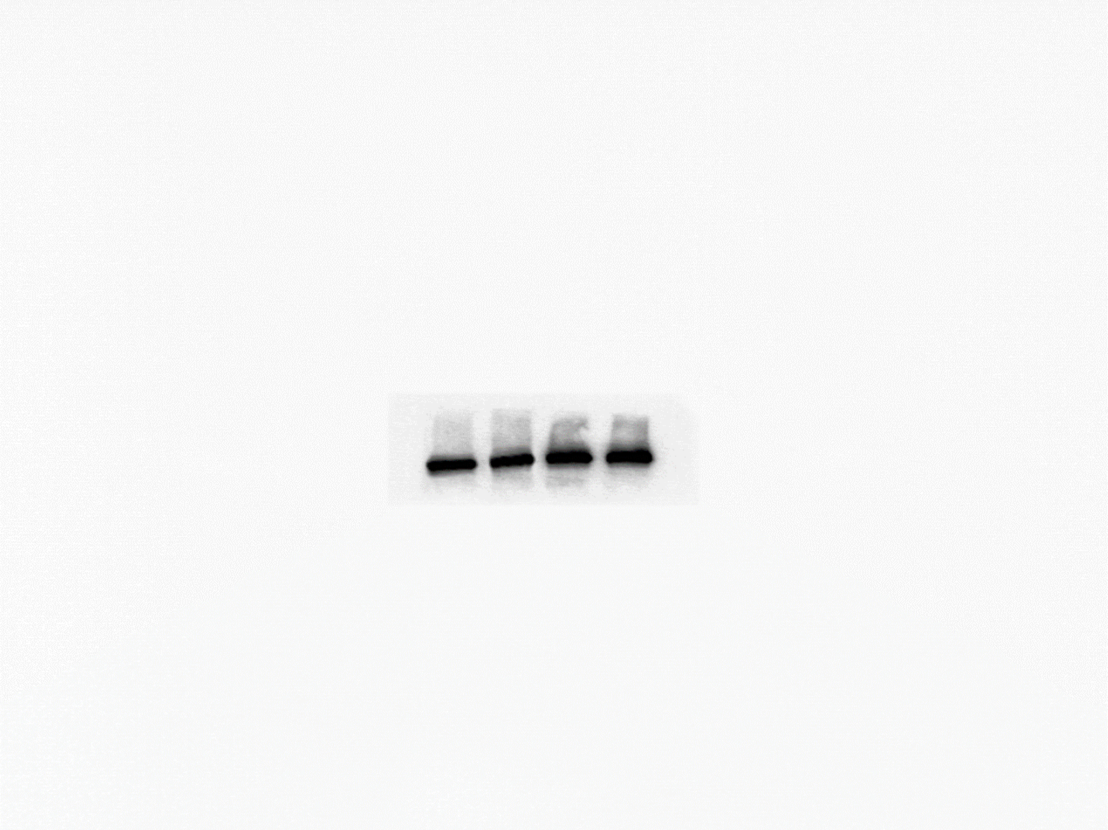

The original gels of Fig.8 in the first experiment:
p-FAK

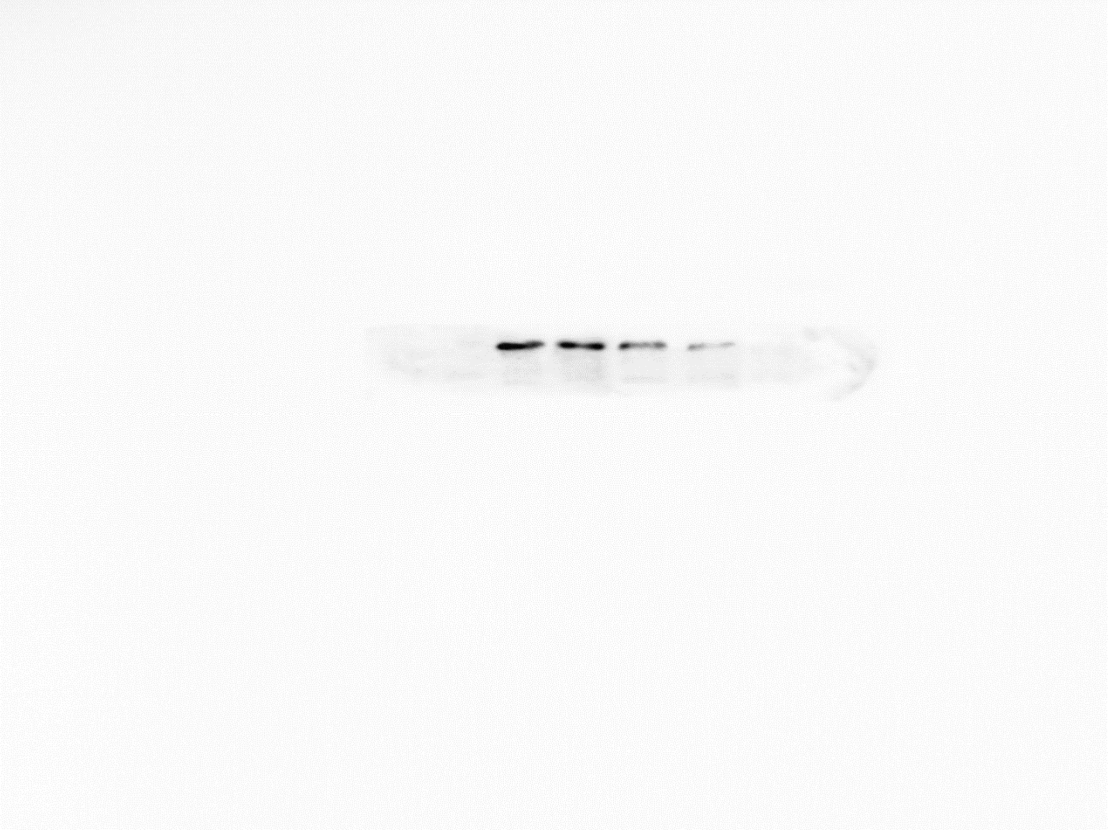

FAK

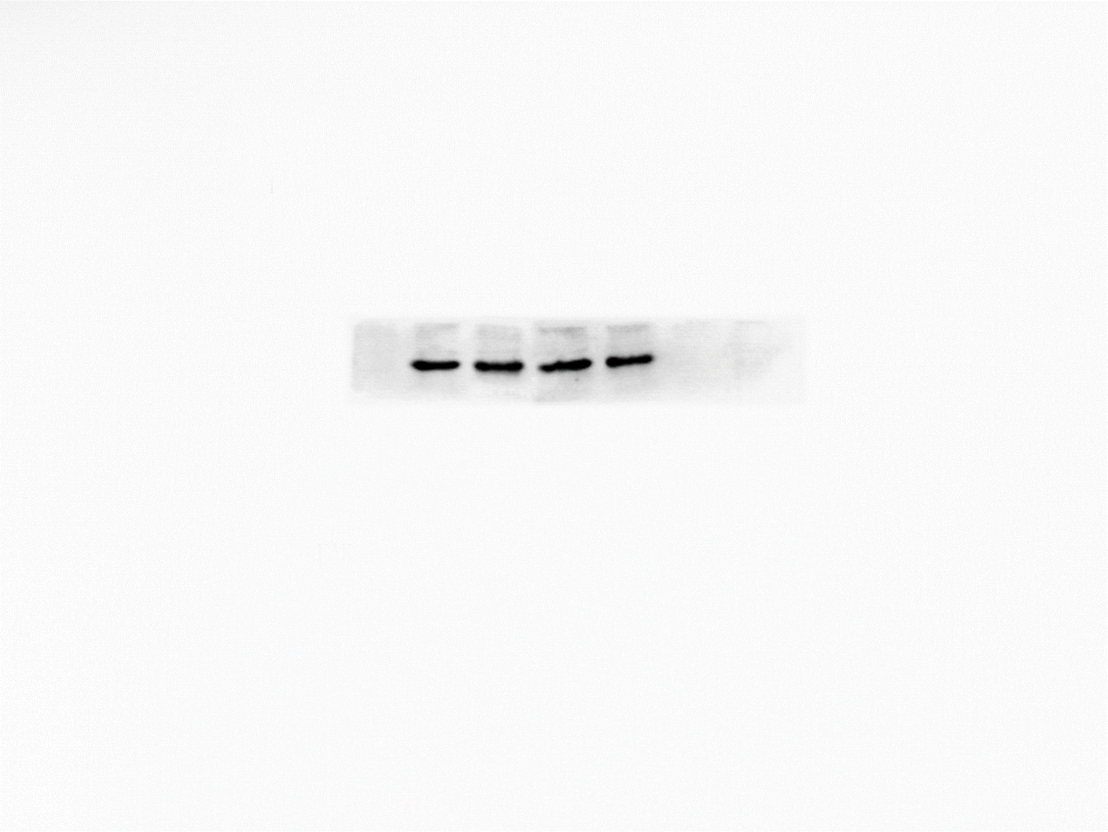

p-PI3K

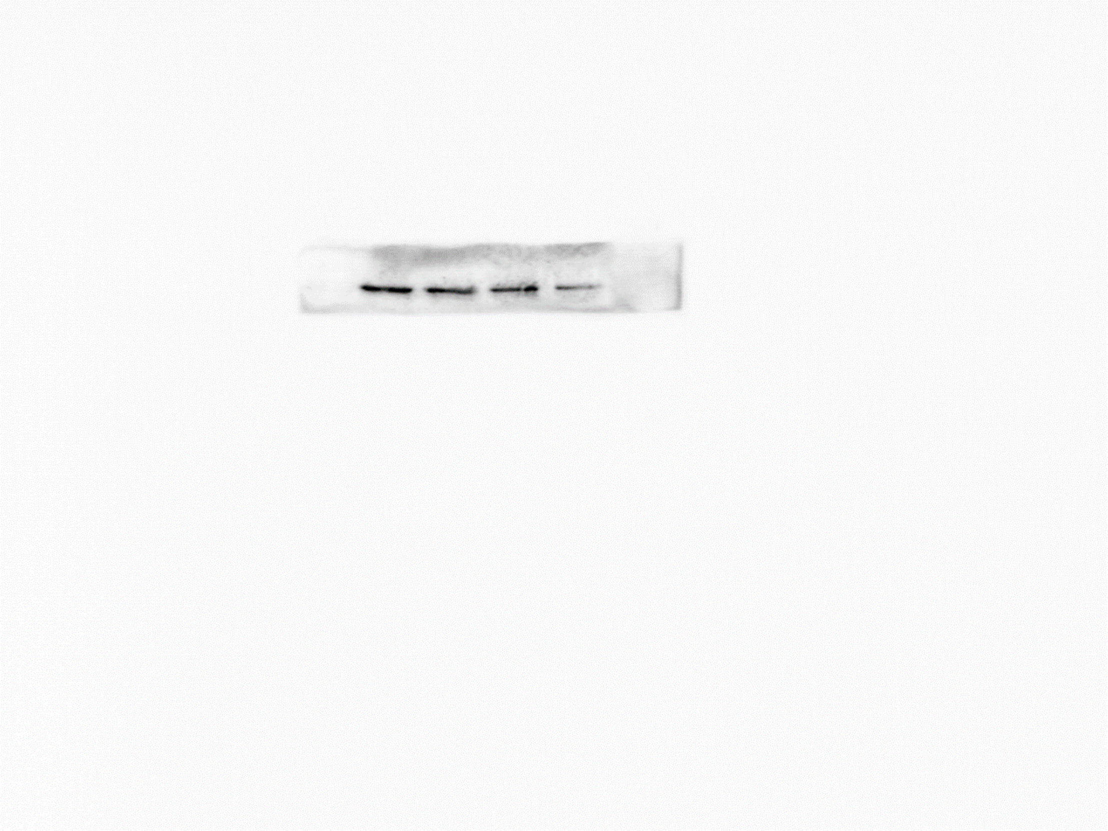

PI3K

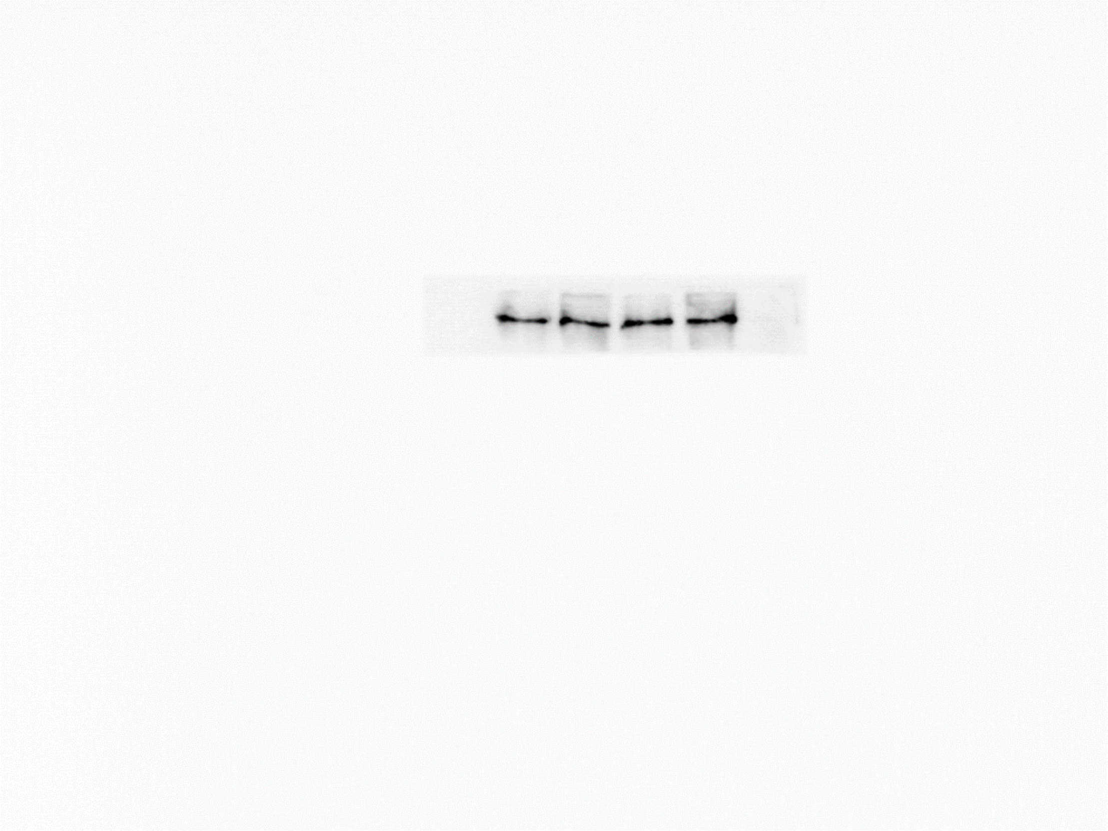

p-AKT

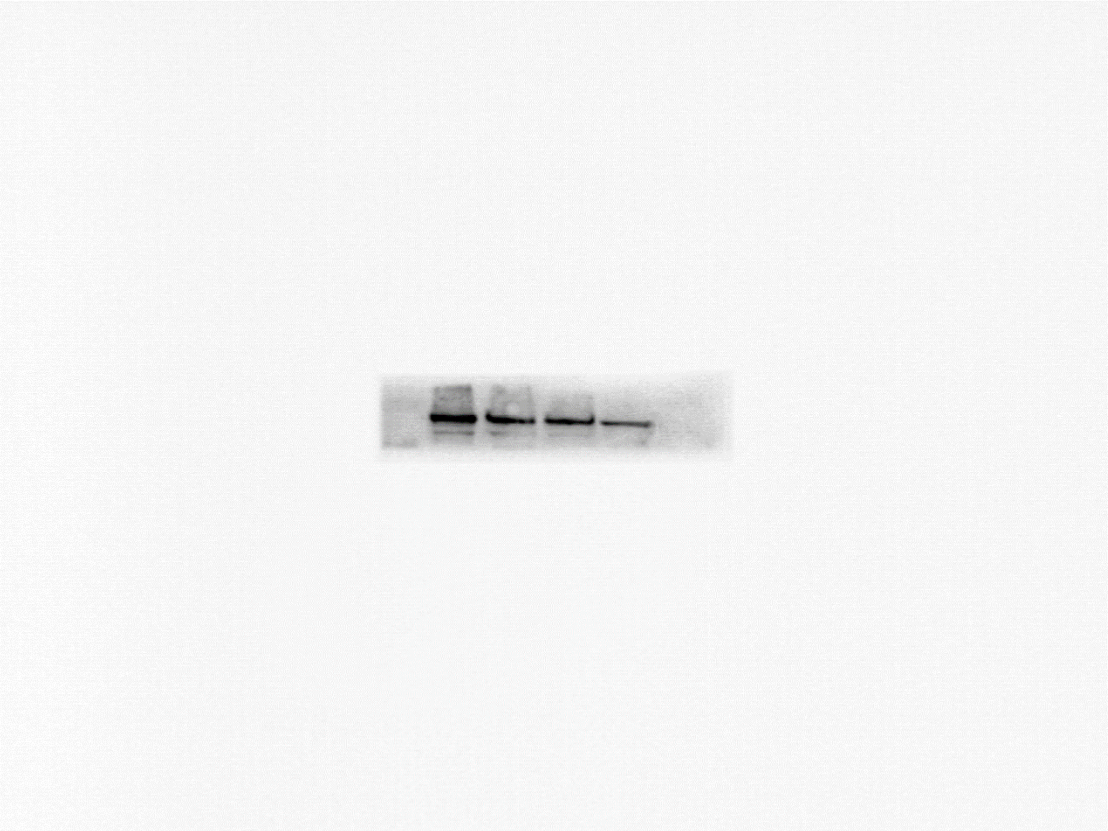

AKT

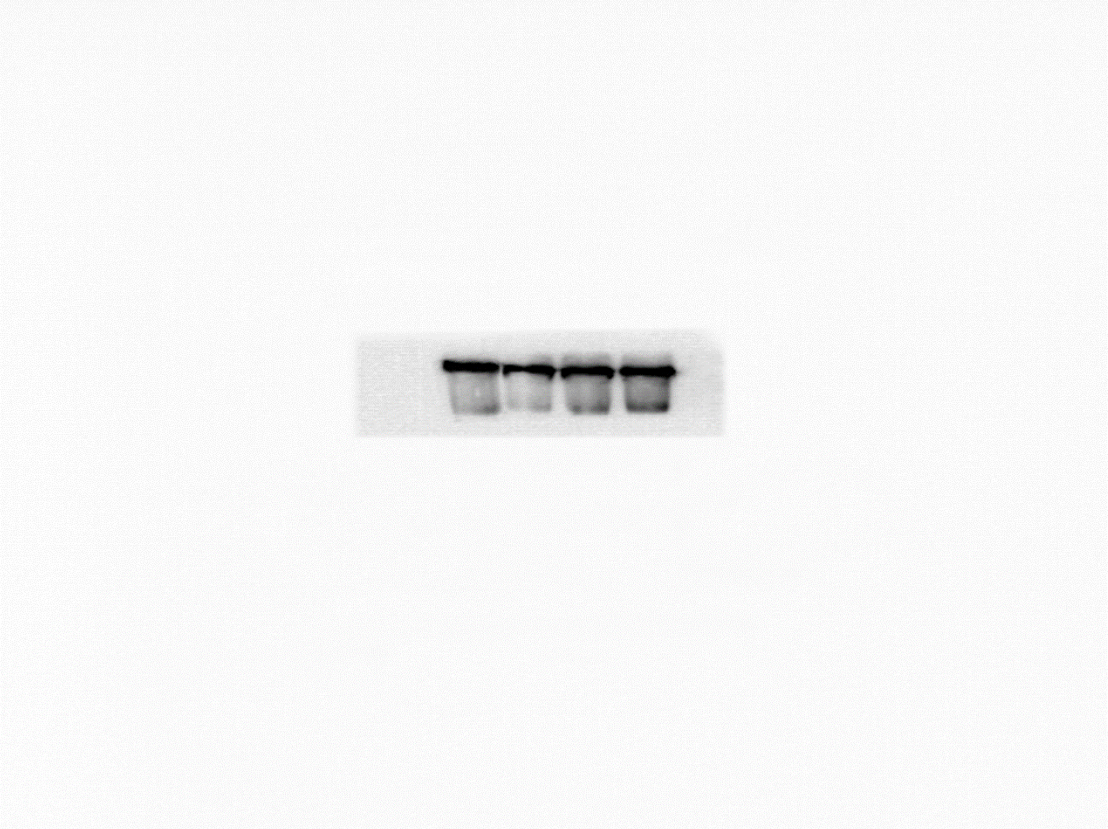

GAPDH

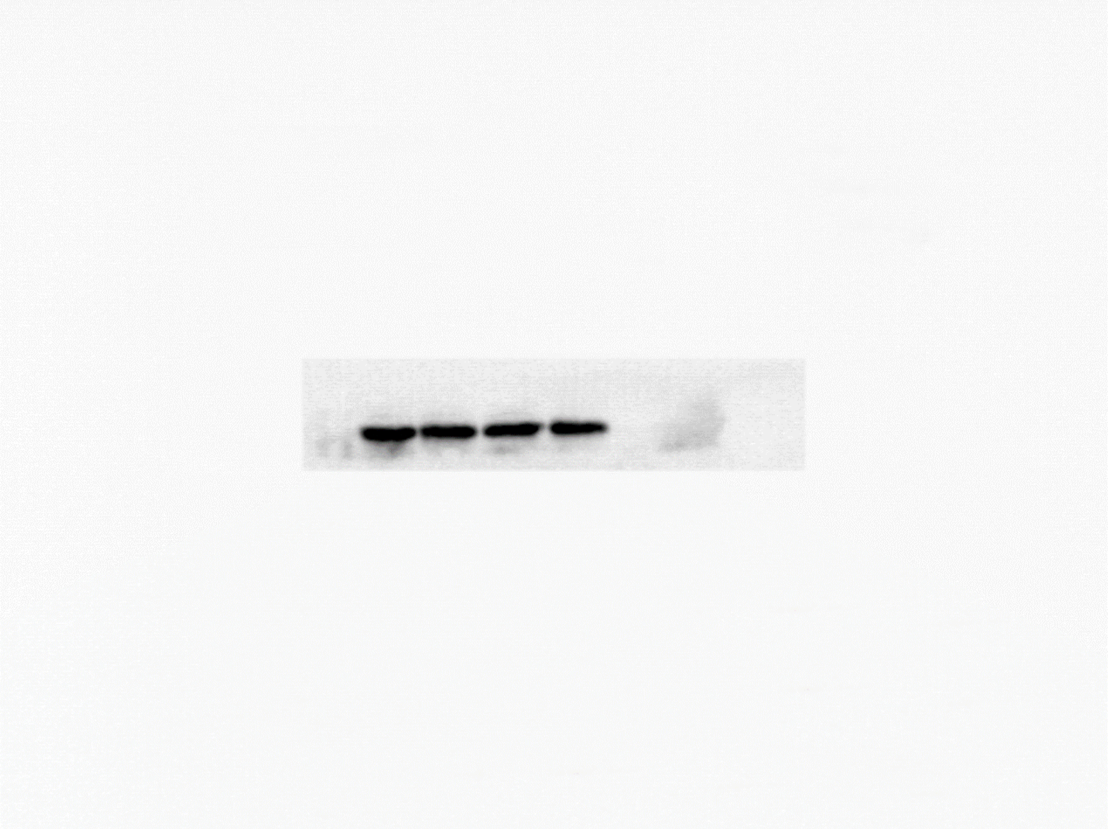

The original gels of Fig.7 and Fig.8 in another two experiments:

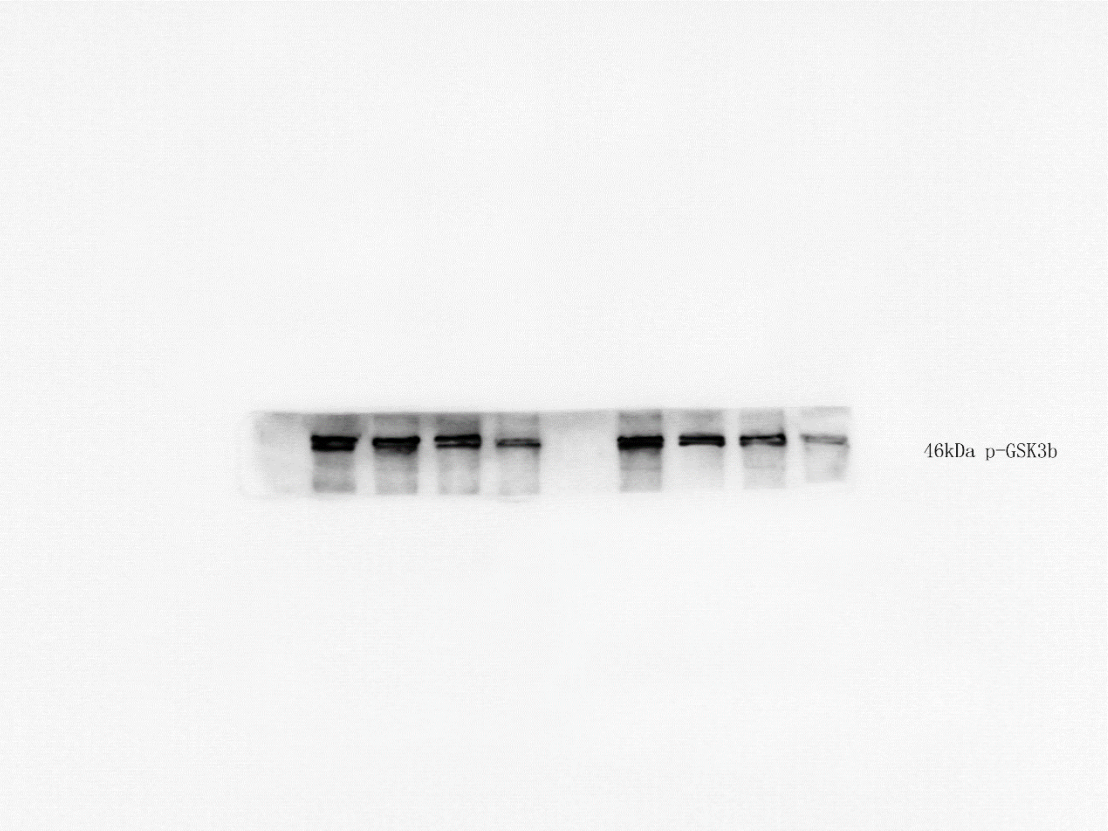

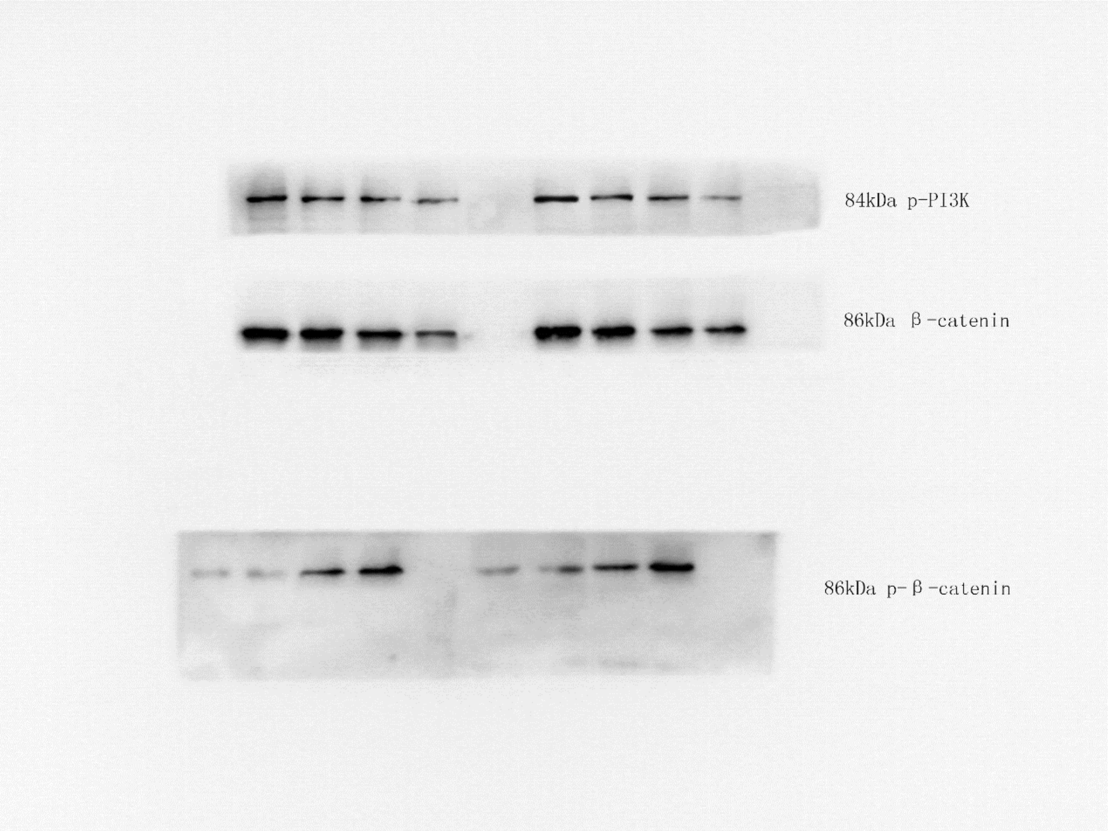

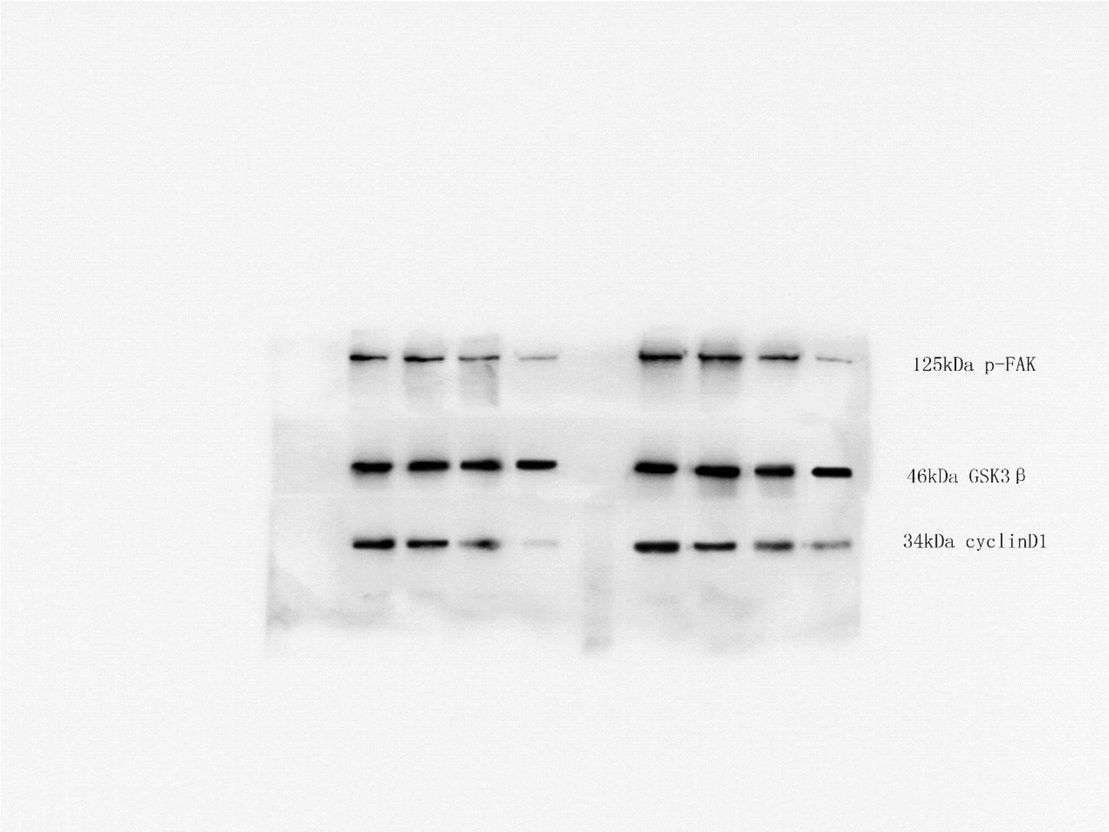
125kDa p-FAK
46kDa GSK3β
34kDa cyclinD1

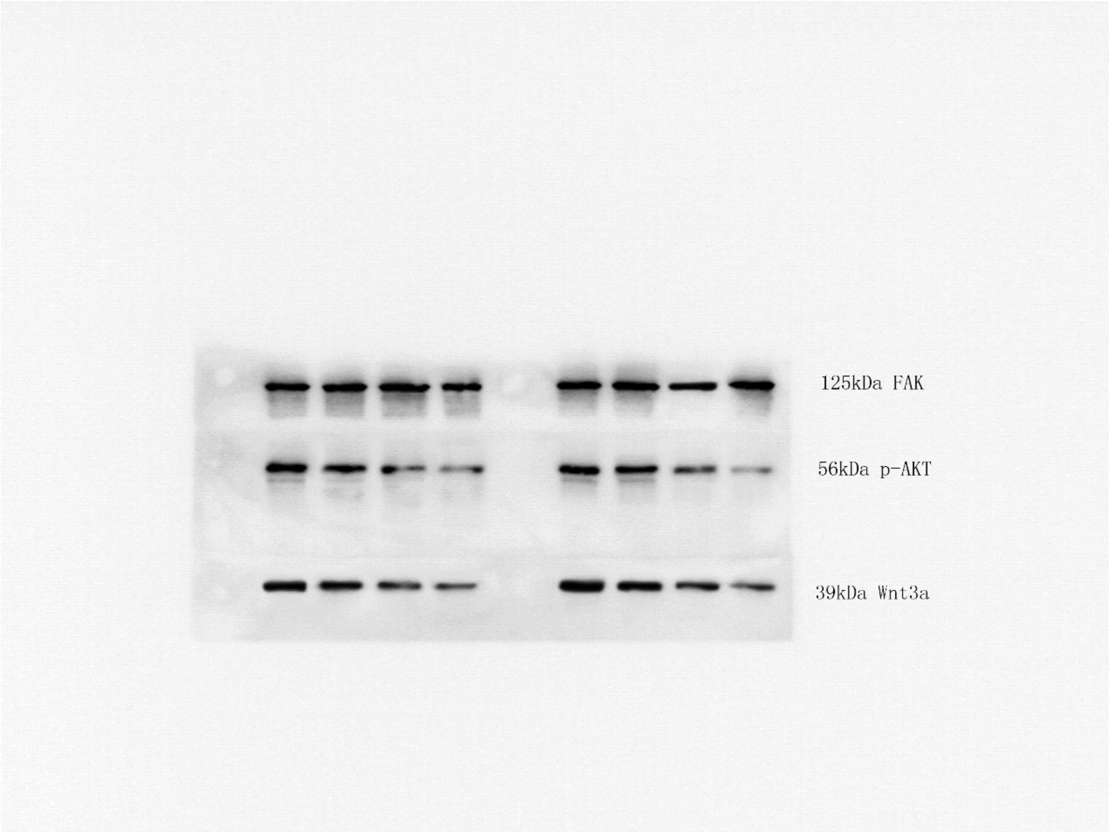
125kDa FAK
56kDa p-AKT
39kDa Wnt3a

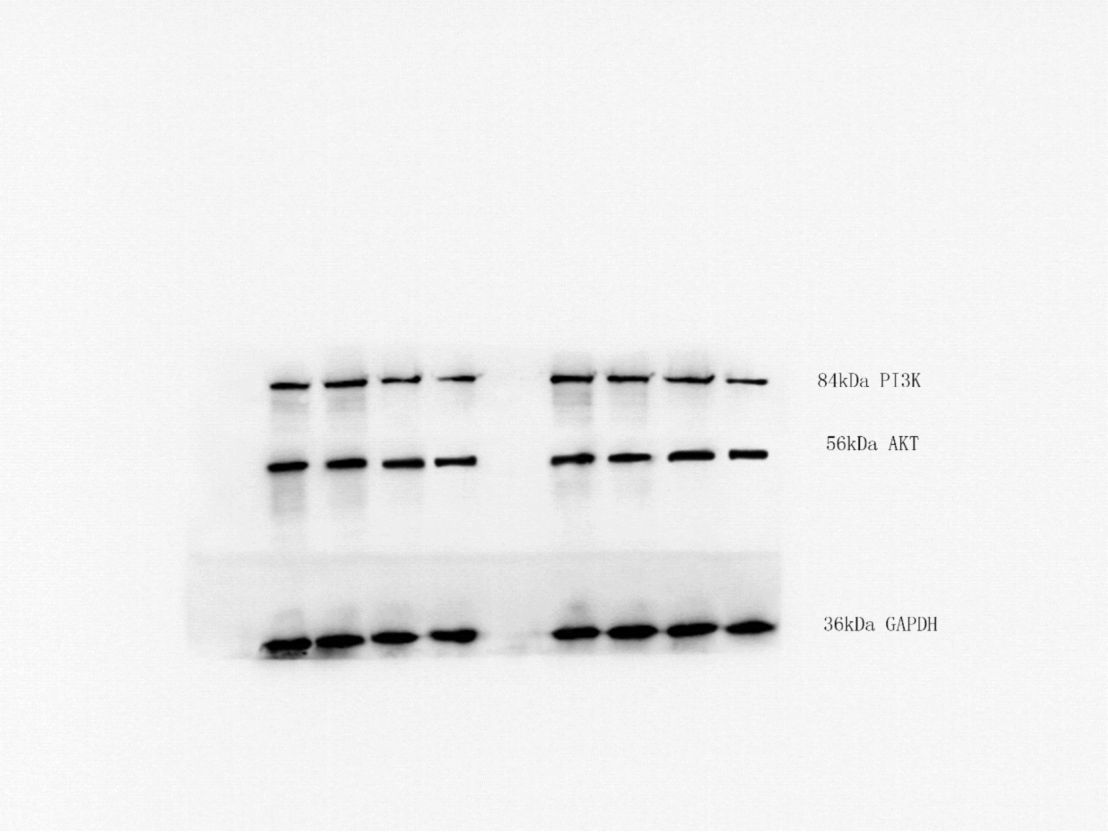
84kDa PI3K
56kDa AKT
36kDa GAPDH
